# Image Encryption Algorithm Based on an Improved ML Neuron Model and DNA Dynamic Coding

**DOI:** 10.1155/2022/4316163

**Published:** 2022-05-14

**Authors:** Yan Cao, Peng Shi, Kaijun Wu, Wenqin Li

**Affiliations:** School of Electronic and Information Engineering, Lanzhou Jiaotong University, Lanzhou 730070, China

## Abstract

Aiming at the problems of small key space, low security, and low algorithm complexity in a low-dimensional chaotic system encryption algorithm, an image encryption algorithm based on the ML neuron model and DNA dynamic coding is proposed. The algorithm first performs block processing on the R, G, and B components of the plaintext image to obtain three matrices, and then constructs a random matrix with the same size as the image components through logistic mapping and performs DNA encoding, DNA operation, and DNA decoding on the two parts. Second, it performs determinant permutation on the matrix by two different chaotic sequences obtained by logistic mapping iteration. Finally, it merges the block and image components to complete the image encryption and obtain the ciphertext image. Wherein, DNA encoding, DNA operation, and DNA decoding methods are all randomly and dynamically determined by the chaotic sequence generated by the ML neuron chaotic system. According to simulation results and performance analysis, the algorithm has a larger key space, can effectively resist various statistical and differential attacks, and has better security and higher complexity.

## 1. Introduction

With the rapid development of cloud computing technology [1] and the widespread application of the 5G network technology [2] in recent years, digital image transmission has already become an indispensable part in all walks of life. As digital images contain a large amount of important data such as security privacy and confidential information, there is a risk of theft in the transmission of digital image information in various fields. Fortunately, the birth of image encryption technology [3–6] solves this problem and ensures the confidentiality of digital images. Since chaotic systems have the characteristics of sensitivity to initial values, pseudo-randomness, and unpredictability, more and more image encryption algorithms based on chaos have been proposed [7–9]. The image encryption algorithm proposed in literature [7] is based on the image segmentation and a multi-diffusion model, which improves the security of the algorithm. The chaotic image encryption algorithm proposed in the literature [8] combines two methods of block scrambling and dynamic index diffusion, and its advantages are fast and safe. The image encryption algorithm based on the fractional order chaotic system proposed in literature [9] has higher security and execution efficiency.

The advantage of DNA computing lies in that it can perform parallel computing, which reduces power consumption and has a high storage density. Researchers have explored the complementary base pairing principle in DNA theory [10–12] and introduced it to realize image encryption. Literatures [13, 14] proposed many DNA-based encryption schemes. Although these schemes combine the chaotic system with DNA encoding theory, which improves the complexity of the system, the security performance still needs improvement because of the not-high chaotic dimension and the not-large key space. In recent years, more and more new image encryption algorithms that combine chaotic mapping and DNA encoding have been proposed by scholars. The newly emerged algorithms have better encryption effects and are more secure than previous ones. Therefore, many researchers from various fields have also begun to pay attention to and deeply understand this type of an image encryption algorithm that combines chaotic mapping and DNA encoding [15–18].

This paper proposes a new image encryption algorithm to generate the required ciphertext image more securely by adopting the DNA random dynamic coding method and combining the ML neuron chaotic system.

## 2. Related Theoretical Knowledge

### 2.1. ML Neuron Model

The ML neuron model is a two-dimensional model [19–21] based on the research results of the muscle fibers of the Arctic goose. This article uses an improved three-dimensional ML neuron model [22, 23], and its differential equation is as follows:

(1) $$\begin{array}{c} \left\{\begin{array}{c} \frac{\text{d}V}{\text{d}t}=-u-g_{l}\left(V-V_{l}\right)-g_{k}\omega \left(V-V_{k}\right)-g_{ca}m_{\infty }\left(V\right)\left(V-V_{ca}\right),\\ \frac{\text{d}\omega }{\text{d}t}=\lambda \left(V\right)\left(\omega_{\infty }\left(V\right)-\omega \right),\\ \frac{\text{d}u}{\text{d}t}=\mu \left(0.2+V\right). \end{array}\right. \end{array}$$

In formula (1): *V* is the neuron membrane potential; *ω* is the neuron recovery variable; *u* is the slow-changing regulation current; *g*_*k*_, *g*_*ca*_, and *g*_*l*_ are the maximum conductance of *K*^+^, *C*a^2+^, and the leakage current channel, respectively; *V*_*k*_, *V*_*ca*_, and *V*_*l*_ are the reversal voltages corresponding to the abovementioned channels, respectively; *λ*(*V*) is the activation time constant; *ω*_*∞*_(*V*) and *m*_*∞*_(*V*) are the steady-state values of the opening probability of the ion channel *K*^+^ and *C*a^2+^, respectively; and *μ* is a constant.

**Figure 1:**
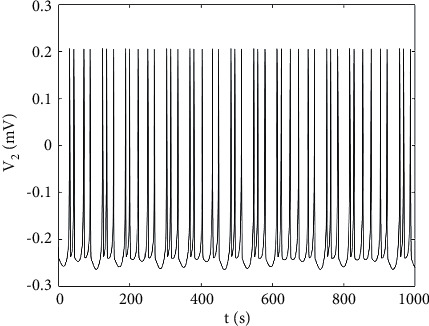
Time history diagram of ML neurons.

**Figure 2:**
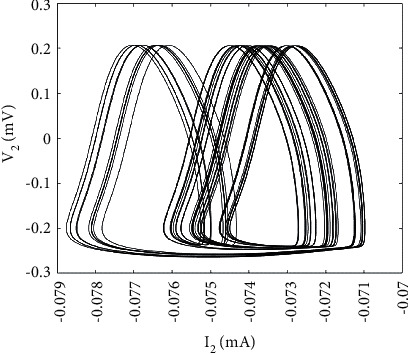
Phase plane diagram of ML neurons.

Part of the parameters of the abovementioned ML neuron model are set, respectively, as *g*_*ca*_ = 1.2 *μ*F/cm^2^, *V*_*ca*_ = 1 mV, *g*_*k*_ = 2 *μ*F/cm^2^, *g*_*l*_ = 0.5 *μ*F/cm^2^, *V*_*l*_ = −0.5 mV, *μ* = 0.005, and keep other parameters unchanged. When the parameter *V*_*ca*_ = 0.83 mV, the ML neuron model is at the chaotic discharge state, whose time history diagram is shown in Figure 1. Moreover, the phase plane diagram also shows that it is in a chaotic state, as shown in Figure 2.

### 2.2. Logistic Chaotic Mapping

The logistic equation is also called the insect-population model [24, 25], and its equation is as formula (2):

(2) $$\begin{array}{c} x\left(n+1\right)=\mu x\left(n\right)\left[1-x\left(n\right)\right], \end{array}$$

wherein *μ* is the system parameter and *x*(*n*) ∈ (0, 1), *μ* ∈ (0, 4). When *μ* ∈ (3.569945, 4), logistic mapping is in a chaotic state, and its dynamic behavior is more complicated.

### 2.3. DNA Encoding and Operations

#### 2.3.1. DNA Encoding

There are 4 kinds of nucleotides in a DNA sequence, namely, A (adenine), G (guanine), C (cytosine), and T (thymine). Wherein, A and T, G, and C have complementary relationships, which are very similar to that of the binary code 0 and 1 in mathematics, so 00 and 11, 01 and 10 also have a complementary relationship. A, T, G, and C are represented by two-digit binary 00, 11, 10, and 01, respectively. According to the principle of complementary pairing, there are 8 sets of encoding rules. As shown in Table 1, the corresponding DNA decoding rules and DNA encoding rules are on the contrary.

#### 2.3.2. DNA Operations

In order to make the algorithm more complex, this paper uses 4 kinds of DNA operations at the same time, namely, DNA addition, DNA subtraction, DNA XOR, and DNA XNOR operations. The operation rules are similar to those of addition, subtraction, XOR, and XNOR in binary mathematics. Because there are 8 DNA encoding methods, so there are 8 corresponding operation rules. Taking one of the DNA encoding methods as an example, the DNA addition, subtraction, XOR, and XNOR operations are shown in Tables 2–5.

## 3. Image Encryption Algorithm

**Figure 3:**
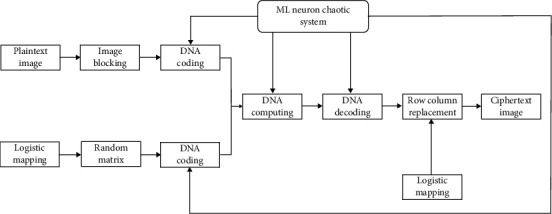
Flowchart of the image encryption algorithm.

This paper divides the color digital image into three two-dimensional matrices, which greatly reduce the time and space resources required. DNA encoding and calculation are performed on each two-dimensional matrix block, then row and column permutations are performed again after encryption, and finally the three channels are merged to obtain a color encrypted image. The three chaotic sequences generated by the ML neuron chaotic system determine the DNA encoding, decoding, and operation rules of each block. The initial value of the chaotic system is determined by the original image to ensure the effect of “one image, one cryptogram.” Three different chaotic sequences which are obtained by performing three iterations on the logistic mapping are used to perform DNA operations with the original image, as well as row and column permutations after DNA decoding operations. The flowchart of the image encryption algorithm is shown in Figure 3. The specific encryption procedure can be summed up by Algorithm 1.

The decryption algorithm is the inverse process of the abovementioned encryption algorithm and will not be described in detail here.

## 4. Experimental Simulation and Safety Performance Analysis

### 4.1. Experimental Simulation

**Figure 4:**
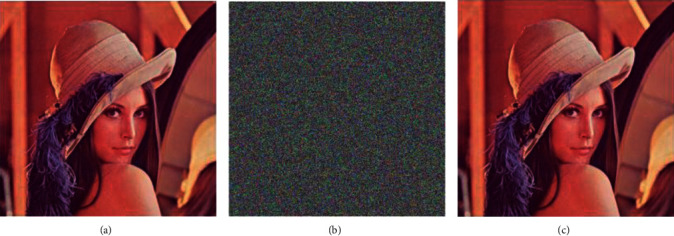
Experimental simulation results. (a) Image before encryption; (b) image after encryption; and (c) image after decryption.

The experiment was completed on the MATLABR2017, a platform in the Win10 system, and the algorithm in this paper was used to encrypt and decrypt a color Lena image with a size of 512 × 512. The result of encrypting and decrypting the Lena image is shown in Figure 4.

### 4.2. Safety Performance Analysis

#### 4.2.1. Histogram Analysis

**Figure 5:**
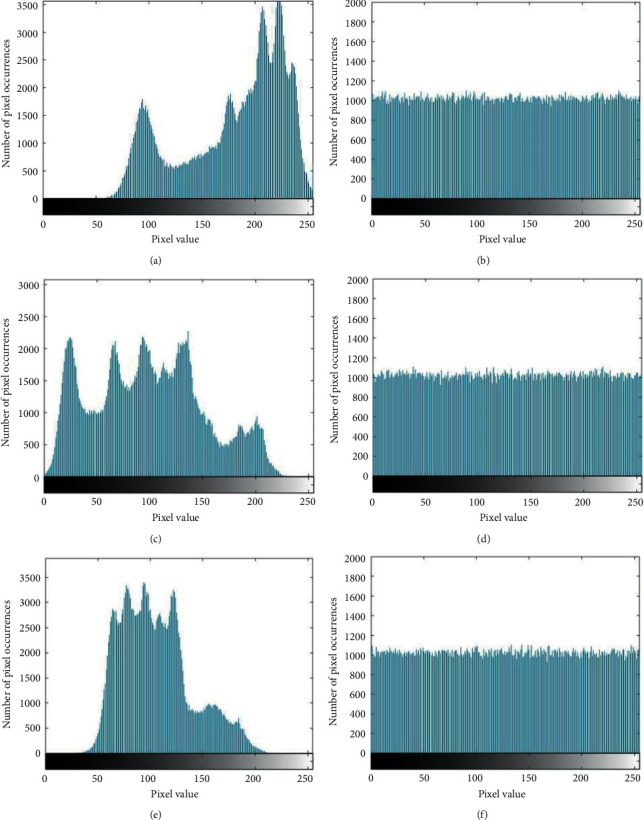
Comparison of histograms before and after image encryption. (a) R channel histogram of the image before encryption; (b) R channel histogram of the image after encryption; (c) G channel histogram of the image before encryption; (d) G channel histogram of the image after encryption; (e) B channel histogram of the image before encryption; and (f) B channel histogram of the image after encryption.

The grayscale histogram of the image can objectively reflect the distribution of image pixels.  Figure 5 shows the grayscale histogram of the R, G, and B channels before and after the Lena image encryption through the comparison of grayscale.

In order to quantitatively compare whether the difference between the histograms of the test ciphertext and the ideal ciphertext image is significant, a chi-square test is used to analyze the uniformity of the histograms. The calculation method is shown in formula (3):

(3) $$\begin{array}{c} \chi^{2}=\sum_{i=1}^{256}\frac{{\left(f_{i}-f\right)}^{2}}{f}, \end{array}$$

wherein, *f*=*M* × *N*/256, *M*, *N* represents the size of the image. *f*_*i*_ is the frequency of occurrence of gray value *i*.

When the significance level is 0.05, the corresponding chi-square critical value is 293.2478. The calculation results of the chi-square analysis are shown in Table 6, from which it can be seen that the chi-square values of all ciphertexts are less than 293.2478, and the encryption algorithm has passed the chi-square test. Therefore, the encryption algorithm proposed in this paper can effectively resist statistical attacks [26].

#### 4.2.2. Correlation Analysis of Adjacent Pixels

The correlation between adjacent pixels of a plaintext image is very strong, so the image is vulnerable to statistical analysis attacks. The calculation formula of the correlation coefficient between two pixels in the image is as shown in formulas (4)–(7):

(4) $$\begin{array}{c} E\left(x\right)=\frac{1}{N}\;\sum_{i=1}^{N}x_{i}, \end{array}$$

(5) $$\begin{array}{c} D\left(x\right)=\frac{1}{N}\;\sum_{i=1}^{N}{\left(x_{i}-E\left(x\right)\right)}^{2}, \end{array}$$

(6) $$\begin{array}{c} \text{cov}\left(x,y\right)=\frac{1}{N}\;\sum_{i=1}^{N}\left(x_{i}-E\left(x\right)\right)\left(y_{i}-E\left(y\right)\right), \end{array}$$

(7) $$\begin{array}{c} r_{xy}=\frac{\mathrm{cov}\left(x,y\right)}{\sqrt{D\left(x\right)D\left(y\right)}}, \end{array}$$

wherein *N* is the logarithm of the required pixel, and *x*, *y* are the gray values of adjacent pixels. Different selection methods of *x*, *y* will lead to the correlation coefficients in different directions, including the horizontal direction, the vertical direction, and the opposite angle direction.

*N* pixels point pairs are randomly selected from different directions, which makes *N* = 5000, and the correlation coefficient of each plaintext and ciphertext is calculated. It can be seen from the results of a certain experiment shown in Table 7 that the correlation coefficients of the three plaintext images in each direction are very large and close to 1, indicating that the adjacent pixels in each direction of the plaintext image are extremely correlated; at the same time, the correlation coefficients of the three ciphertext images are all extremely small and very close to 0, indicating that the correlation between adjacent pixels in all directions of the ciphertext image is extremely weak. In addition, compared with the methods proposed in literature [27] and literature [28], the algorithm proposed in this paper can eliminate the correlation between adjacent pixels and conceal the data characteristics of the original image.

**Figure 6:**
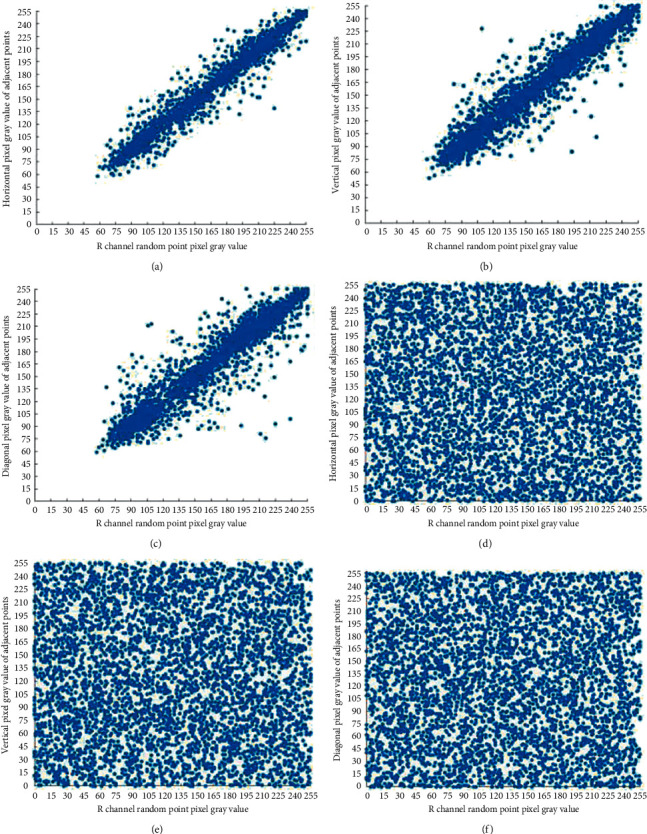
Adjacent pixels correlation of the R channel before and after encryption. (a) Horizontal correlation of the original image R channel; (b) vertical correlation of the original image R channel; (c) diagonal correlation of the original image R channel; (d) horizontal correlation of the ciphertext image R channel; (e) vertical correlation of the ciphertext image R channel; and (f) diagonal correlation of the ciphertext image R channel.

**Figure 7:**
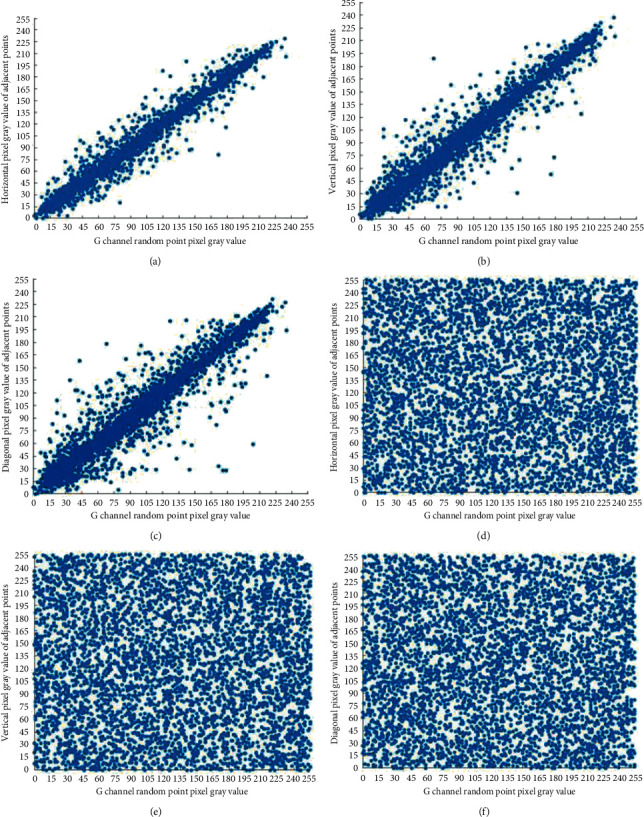
Adjacent pixels correlation of the G channel before and after encryption. (a) Horizontal correlation of the original image G channel; (b) vertical correlation of the original image G channel; (c) diagonal correlation of the original image G channel; (d) horizontal correlation of the ciphertext image G channel; (e) vertical correlation of the ciphertext image G channel; and (f) diagonal correlation of the ciphertext image G channel.

**Figure 8:**
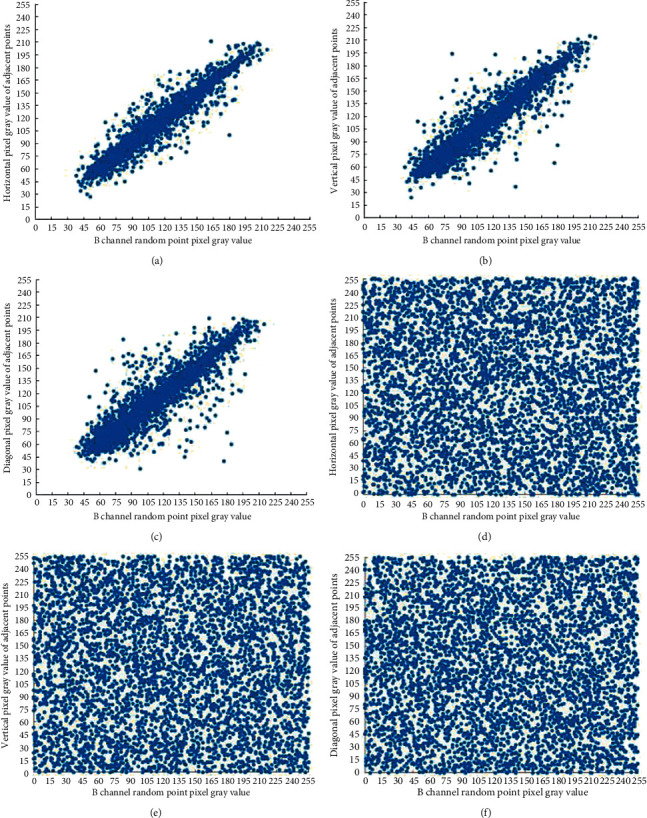
Adjacent pixels correlation of the B channel before and after encryption. (a) Horizontal correlation of the original image B channel; (b) vertical correlation of the original image B channel; (c) diagonal correlation of the original image B channel; (d) horizontal correlation of the ciphertext image B channel; (e) vertical correlation of the ciphertext image B channel; and (f) diagonal correlation of the ciphertext image B channel.

Figures 6–8are the pixel distribution diagrams of three channels R, G, and B of the Lena plaintext and ciphertext image in different directions. It can be seen that the distribution of plaintext pixels is linear, while the distribution of ciphertext pixels is disorderly and irregular. Therefore, the algorithm in this paper can effectively reduce the correlation between adjacent pixels of plaintext images and can effectively resist statistical analysis attacks.

#### 4.2.3. Global Entropy and Local Entropy Analysis

The definition of information entropy refers to the uncertainty of information, while the definition of image information entropy refers to the distribution probability of image gray values. When the distribution probability reaches the equal condition, the information entropy will be at the maximum value, which shows that the randomness of the ciphertext image is very high and that the encryption algorithm has a strong ability to resist statistical attacks. The calculation formula of global entropy is as formula (8):

(8) $$\begin{array}{c} H\left(m\right)=-\sum_{i=1}^{L}p\left(m_{i}\right)\log_{2}\;p\left(m_{i}\right), \end{array}$$

where *L* refers to the gray level of the image and *p*(*m*_*i*_) represents the probability that the gray value *m*_*i*_ appears.

Table 8 shows the comparison with the encryption algorithms in existing literature. It can be seen that the global entropy of the encryption algorithm proposed in this paper is closer to the ideal value of 8.

On the basis of global entropy, Wu Y et al. proposed a calculation method called “local Shannon entropy,” which overcomes the shortcomings of inaccuracy, inconsistency, and low efficiency of global entropy [31]. The local entropy is an improvement over the global entropy. It first randomly selects non-overlapping blocks in the image and then calculates the average value of the global entropy for the small image blocks. According to the method described in the literature, 30 non-overlapping blocks are randomly selected in the image. When the number of pixels in each block is 1936, the local entropy is calculated, and the ideal value is 7.9024. Within the 0.01 level, if the local entropy is between 7.9017 and 7.9032, it means that the local entropy of the image is ideal and the image has good randomness. The local entropy test results are shown in Table 9.

#### 4.2.4. Key Sensitivity Analysis

Key sensitivity refers to the resulting chaotic sequence changes after a small change to the key in the encryption algorithm, which has a great impact on the encrypted and decrypted image. If the key is not sensitive enough to correctly reconstruct the original image even with a slightly different key, the key may degenerate. In this paper, the key sensitivity test method of the encryption algorithm is given.

**Figure 9:**
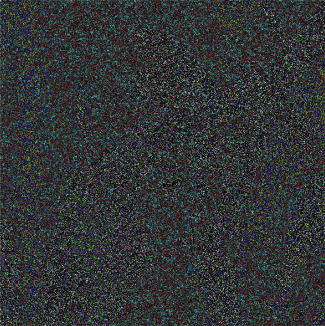
Decrypted image when the key is wrong.

The first method is direct observation. The same encrypted image is decrypted with a slightly changed key and the differences between the images are observed. We can improve the anti-cracking ability of the encryption algorithm by increasing a certain number of digits after the decimal point of the key. The decrypted image obtained when one of the encrypting keys *x*_0_ is changed from 0.5475 to 0.547500000000001 is shown in Figure 9. It can be found that the decryption is not successful when the key is changed slightly, which shows that the proposed encryption algorithm has good key sensitivity and can effectively resist the exhaustive attacks.

**Figure 10:**
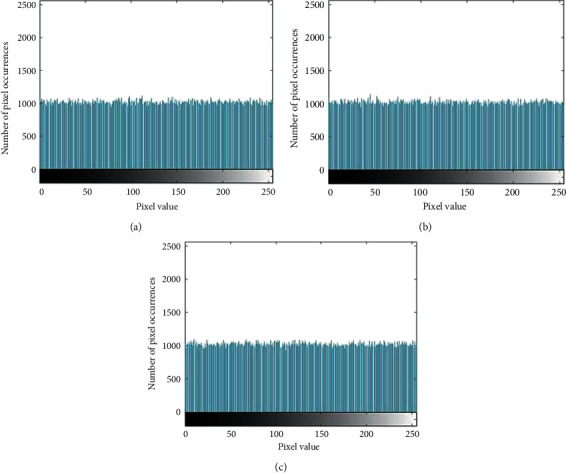
Histogram of the incorrectly decrypted image. (a) Histogram of the R channel; (b) histogram of the G channel; and (c) histogram of the B channel.

When one of the keys changes slightly, the histograms of the three channels of R, G, and B of the incorrectly decrypted images are as shown in Figure 10. It can be found that even if the original image is not successfully decrypted, the histograms of the decrypted image are still evenly distributed and can effectively resist attacks.

The second method is to use the indicator NBCR (number of bit change rate) to quantitatively illustrate the key sensitivity from another perspective [32]. The NBCR of matrices *B*_1_ and *B*_2_ is defined as formula (9):

(9) $$\begin{array}{c} \text{NBCR}\left(B_{1},B_{2}\right)=\frac{\text{Ham}\left(B_{1},B_{2}\right)}{\text{len}}, \end{array}$$

where Ham(*B*_1_, *B*_2_) is the Hamming distance of the two matrices *B*_1_ and *B*_2_ and len is the total number of bits of *B*_1_ and *B*_2_.

If the NBCR result is close to 50%, it means that *B*_1_ and *B*_2_ are completely different matrices without any correlation. Specifically, the seven parameters (*μ*, *x*_0_, *x*_01_, *x*_02_, *X*(0), *Y*(0), *Z*(0)) in the algorithm are used as keys to form an infinite dimensional key space, and *K* is slightly changed (10^−11^) to obtain another key *K*′. Every parameter change in (*μ*, *x*_0_, *x*_01_, *x*_02_, *X*(0), *Y*(0), *Z*(0)) is in a very small *δ* interval. For example, the small change of *x*_0_ is *x*_0_+10^−11^ ∈ [*x*_0_ − *δ*, *x*_0_+*δ*](*δ*=10^−10^), and other parameters remain unchanged. Encrypting the original image with the keys *K* and *K*′, respectively, to obtain the encryption results *C*_1_ and *C*_2_, and then calculate the NBCR values of *C*_1_ and *C*_2_; the same encryption result is decrypted with keys *K* and *K*′, respectively, to obtain decryption results *D*_1_ and *D*_2_, and then calculate the NBCR values of *D*_1_ and *D*_2_. The NBCR test results of the algorithm in this paper are shown in Table 10.

It can be seen from Table 10 that the calculated NBCR is very close to 50%, indicating that the results are completely different, which means that the algorithm is very sensitive to slightly changed keys.

#### 4.2.5. Plaintext Sensitivity Analysis

The plaintext sensitivity analysis aims to analyze the degree of change of the ciphertext image caused by the slight change of the plaintext image under the same key condition. When the change is large, the encryption system has a strong plaintext sensitivity and can effectively resist differential attacks. NPCR (pixel change rate) and UACI (pixel average change intensity) are usually used to detect the ability of image encryption schemes to resist differential attacks. The calculation formulas (10) and (11) are as follows:

(10) $$\begin{array}{c} \text{NPCR}=\frac{\sum_{m=1}^{M}\sum_{n=1}^{N}C\left(m,n\right)}{M\times N}\times 100\%, \end{array}$$

(11) $$\begin{array}{c} \text{UACI}=\frac{\sum_{m=1}^{M}\sum_{n=1}^{N}\left|I_{1}\left(m,n\right)-I_{2}\left(m,n\right)\right|}{M\times N\times 255}\times 100\%. \end{array}$$

In the formula, *M* × *N* is the image size. Assuming that two plaintext images are different in only one pixel, after using the same algorithm to encrypt, the pixel values of the positions (*m*, *n*) in the ciphertext image are, respectively, *I*_1_(*m*, *n*) and *I*_2_(*m*, *n*); when the value of the two is the same, then the value of *C*(*m*, *n*) is 0, otherwise the value is 1. In the Lena plaintext, a pixel is randomly selected and its value is increased by 1, the same algorithm is used to encrypt, and the NPCR and UACI values are calculated. The results are shown in Table 11.

It can be seen from Table 11 that the NPCR of the image encrypted by the algorithm proposed in this paper is close to the ideal value of 100%, and the UACI is close to the ideal value of 33%. Compared with literature [33] and literature [34], the algorithm in this paper has stronger anti-differential attack capability.

Although the calculated NPCR and UACI values are close to the ideal values, only the NPCR and UACI values of the encrypted image are given quantitatively. In order to qualitatively analyze whether the encryption algorithm can produce ciphertext images that are secure enough and can resist differential attacks, Wu et al. proposed the method of randomness test for NPCR and UACI, analyzed their confidence intervals, and obtained critical value results under different parameter combinations. For detailed results, please refer to reference [35].

At the same time, two images of different sizes are taken. According to the abovementioned method, the NPCR and UACI values of the two ciphertext images are obtained and compared with the corresponding critical values (*α* = 0.05). The test results are shown in Table 12. From the data in the table, it can be seen that the NPCR value and the UACI value are both within the confidence interval, so it can be proved that the algorithm proposed in this paper is sufficient to resist differential attacks.

#### 4.2.6. Key Space Analysis

The algorithm proposed in this paper uses a total of three logistic chaotic mappings. Although the values of parameter *μ* are the same, the initial values *x*_0_ of each time are different. Therefore, *μ*, *x*_0_, and the three initial values *X*(0), *Y*(0), and *Z*(0) of the ML neuron chaotic system can all be used as the system keys. The image is decrypted by changing the number of digits after the decimal point of the key to get the key sensitivity 10^−15^, 10^−16^, 10^−16^, 10^−16^, 10^−16^, 10^−16^, 10^−16^ of the key *μ*, *x*_0_, *x*_01_, *x*_02_, *X*(0), *Y*(0), *Z*(0), respectively, so the key capacity 10^15^ × 10^16^ × 10^16^ × 10^16^ × 10^16^ × 10^16^ × 10^16^=10^111^ of the encryption algorithm is obtained. Literature [36] pointed out that the key space of a security cryptosystem should be greater than 2^100^ and both ENISA and NIST standards require a key size of at least 2^112^. The key space of the encryption scheme in this paper is larger than the above standards.

#### 4.2.7. Robustness Analysis

This paper uses noise attack and cropping attack to analyze the robustness of the proposed image encryption algorithm. There is often a lot of noise in the transmission channel, which has a serious impact on the transmission of encrypted images, and even causes the decryption algorithm to fail to restore the identifiable original image. Therefore, an excellent encryption algorithm must have certain noise robustness. In the process of information transmission, the loss of encrypted image information data will occur from time to time, which is often referred to as cropping attack. Therefore, the image encryption algorithm needs to be able to resist the cropping attack, so that it can successfully decrypt and recover recognizable images. PSNR (peak signal-to-noise ratio) between the original image and the decrypted image is an important indicator to measure the quality of the decrypted image, and its definition is shown in formulas (12) and (13).

(12) $$\begin{array}{c} \text{MSE}=\frac{1}{M\times N}\sum_{i=1}^{M}\sum_{j=1}^{N}{\left(P\left(i,j\right)-D\left(i,j\right)\right)}^{2}, \end{array}$$

(13) $$\begin{array}{c} \text{PSNR}=10\times \;\log\frac{{\text{MAX}}_{I}^{2}}{\text{MSE}}, \end{array}$$

where *M* × *N* is the image size, *P*(*i*, *j*) and *D*(*i*, *j*) are the original image and decrypted image, respectively, and MAX_*I*_^2^ is the square of the maximum pixel value of the image.

**Figure 11:**
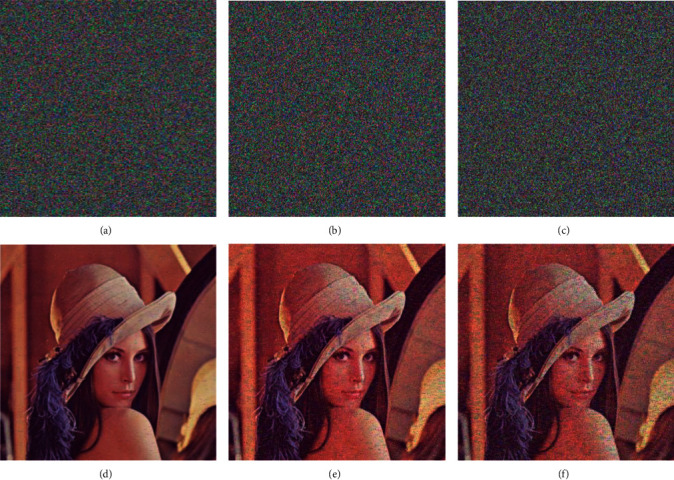
Decryption effect of encrypted image after adding salt and pepper noise. (a) Add 0.005 salt and pepper noise; (b) add 0.05 salt and pepper noise; (c) add 0.1 salt and pepper noise; (d) decrypted image with salt and pepper noise of 0.005; (e) decrypted image with salt and pepper noise of 0.05; and (f) decrypted image with salt and pepper noise of 0.1.

In order to analyze the anti-noise capability of the encrypted image, salt and pepper noises with different noise densities were added to the encrypted image, and the PSNR of the original image and the decrypted image was calculated. The Lena encrypted images after adding noise densities of 0.005, 0.05, and 0.1 and the images decrypted using the proposed encryption algorithm are shown in Figure 11. It can be concluded from the figure that under the attack of salt and pepper noise, the decrypted image can still be recognized well. The PSNR value between the original image and the decrypted image under the noise attack is calculated , and the results are shown in Table 13. Experiments show that the proposed encryption algorithm has strong robustness against salt and pepper noise attacks.

**Figure 12:**
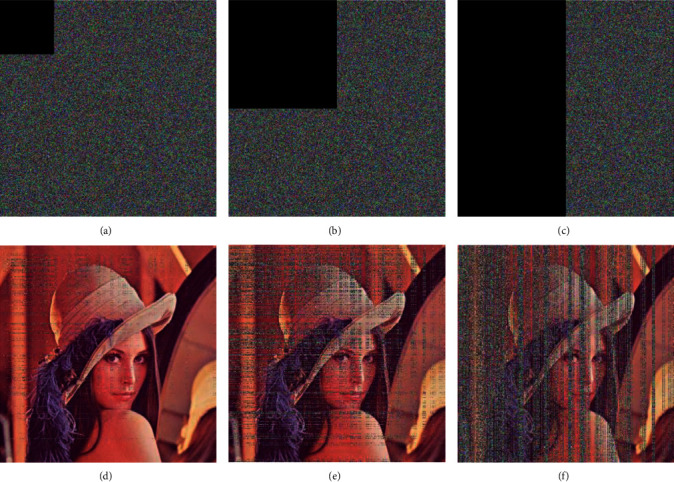
Decryption effect after the encrypted image part is cropped. (a) Crop 1/16 of the encrypted image; (b) crop 1/4 of the encrypted image; (c) crop 1/2 of the encrypted image; (d) decrypted image when cropped by 1/16; (e) decrypted image when cropped by 1/4; and (f) decrypted image when cropped by 1/2.

In order to analyze the anti-cropping attack capability of the encrypted image, the data of the encrypted image are crop by 1/16, 1/4 and ½, respectively, and the encryption algorithm proposed in this paper is used for decryption, respectively. The experimental effect is shown in Figure 12. As can be seen from the figure, under the crop attack, the decrypted image still has a certain degree of recognition. The PSNR value between the original image and the decrypted image under the crop attack is calculated, and the results are shown in Table 14. Experiments show that the proposed encryption algorithm has the ability to resist crop attack.

#### 4.2.8. Speed Performance Analysis

In addition to considering security, encryption speed is also an important aspect to measure the pros and cons of encryption algorithms, especially for real-time network transmission requirements. For images of different sizes, the encryption speed of this algorithm and existing encryption algorithms is compared, as shown in Table 15. The computer used for testing is Intel(R) Core(TM) i5-8265U CPU (1.60 GHz), 8G memory. It can be seen from the table that the algorithm in this paper has certain advantages and high real-time performance, which is very necessary for the image encryption algorithm.

## 5. Conclusions

The encryption algorithm proposed in this paper combines an improved ML neuron model with DNA encoding. First, the original image and the logistic chaotic matrix are divided into blocks and DNA encoding is performed, respectively, and then the DNA operation and decoding between the same positions of the two image blocks is continued. Second, the continuous iteration of the chaotic sequence is used to perform determinant permutation on the matrix. Finally, the ciphertext image is obtained, in which the DNA encoding, operation, and decoding methods are determined by the chaotic sequence generated by the ML neuron chaotic system. Encryption performance analysis proves that the algorithm has a larger key space, better statistical characteristics, stronger key sensitivity and plaintext sensitivity, and can effectively resist various attacks such as exhaustive attacks, statistical analysis attacks, and differential attacks, indicating that it has good security and effectiveness.

## Figures and Tables

**Algorithm 1:**
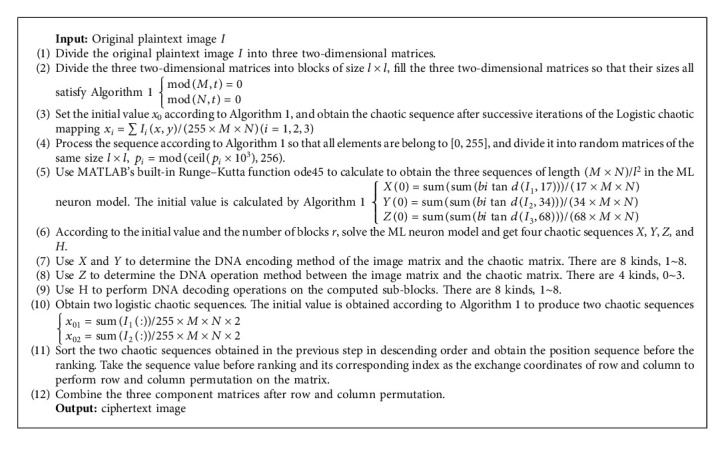
The image encryption algorithm.

**Table 1:** DNA encoding table.

	1	2	3	4	5	6	7	8
00	A	A	C	C	G	G	T	T
01	C	G	A	T	A	T	C	G
10	G	C	T	A	T	A	G	C
11	T	T	G	G	C	C	A	A

**Table 2:** DNA addition.

	A	C	G	T
A	A	C	G	T
C	C	G	T	A
G	G	T	A	C
T	T	A	C	G

**Table 3:** DNA subtraction.

	A	C	G	T
A	A	T	G	C
C	C	A	T	G
G	G	C	A	T
T	T	G	C	A

**Table 4:** DNA XOR.

	A	C	G	T
A	A	C	G	T
C	C	A	T	G
G	G	T	A	C
T	T	G	C	A

**Table 5:** DNA XNOR.

	A	C	G	T
A	T	G	C	A
C	G	T	A	C
G	C	A	T	G
T	A	C	G	T

**Table 6:** Chi-square test results.

	*χ*_test_^2^	*χ*_256,0.05_^2^	Test results
Ciphertext image R channel	268.1753	293.2478	Pass
Ciphertext image G channel	259.2314	293.2478	Pass
Ciphertext image B channel	263.1372	293.2478	Pass

**Table 7:** Correlation coefficients of adjacent pixels and comparison between different algorithms.

Direction		Plaintext	Ciphertext	Literature [27]	Literature [28]
Level	R	0.97716	−0.012388	0.0137	0.0049
	G	0.97731	0.0064409	−0.0246	−0.0054
	B	0.9556	−0.0041111	−0.0137	0.0053

Vertical	R	0.98774	−0.017358	−0.0237	0.0031
	G	0.98834	−0.0001329	−0.0170	0.0001
	B	0.97433	−0.025994	0.0023	0.0022

Diagonal	R	0.96407	0.016901	0.0109	0.0007
	G	0.96433	−0.0064608	0.0133	−0.0017
	B	0.93203	0.002631	−0.0013	−0.0007

**Table 8:** Comparison of global entropy of each channel before and after image encryption.

	R channel	G channel	B channel
Original image	7.2682	7.5901	6.9951
Ciphertext image	7.9994	7.9993	7.9993
Literature [29]	7.9971	7.9974	7.9973
Literature [30]	7.9914	7.9915	7.9916

**Table 9:** Local information entropy test.

	Local information entropy	Test results
Ciphertext image R channel	7.9023	Pass
Ciphertext image G channel	7.9019	Pass
Ciphertext image B channel	7.9027	Pass

**Table 10:** Test results of NBCR (%).

Key	NBCR (*C*_1_, *C*_2_)	NBCR (*D*_1_, *D*_2_)
*μ*	49.9967	50.0014
*x*_0_	50.0021	49.9956
*x*_01_	50.0039	49.9996
*x*_02_	49.9983	50.0044
*X*(0)	49.9989	49.9997
*Y*(0)	50.0012	50.0029
*Z*(0)	49.9961	50.0024

**Table 11:** Comparison of pixel change rate and average change intensity of each literature using the same image.

	NPCR (%)	UACI (%)
Paper algorithm	99.61	33.44
Literature [33]	99.55	33.44
Literature [34]	99.60	33.40

**Table 12:** NPCR and UACI test (*α*  = 0.05).

Test image	Image size	Test content	Calculation results (%)	Confidence interval test
Cameraman	256 × 256	NPCR	99.59	Pass
Cameraman	256 × 256	UACI	33.35	Pass
Lena	512 × 512	NPCR	99.61	Pass
Lena	512 × 512	UACI	33.44	Pass

**Table 13:** PSNR of the original image and decrypted image under noise attack.

Test image	Noise density	Paper algorithm	Literature [37]	Literature [38]
Lena	0.005	30.2474	31.4161	29.9684
	0.05	20.3605	21.5482	19.9800
	0.1	17.8897	18.5665	17.0283

**Table 14:** PSNR of the original image and decrypted image under crop attack.

Test image	Crop	Paper algorithm	Literature [37]	Literature [38]
Lena	1/16	20.0312	20.0200	20.0820
	1/4	14.2157	14.6140	14.3566
	1/2	11.2968	11.8632	11.4038

**Table 15:** Encryption speed comparison of images of different sizes (s).

Image size	Paper algorithm	Literature [39]	Literature [40]
256 × 256	0.4282	0.2736	0.4389
512 × 512	1.7967	1.6166	1.8112

## Data Availability

The data used to support the findings of this study are available from the corresponding author upon request.
